# The Impact of the COVID-19 Pandemic on Pregnancy Planning Behaviors

**DOI:** 10.1089/whr.2021.0005

**Published:** 2021-03-23

**Authors:** Angela C. Flynn, Kimberley Kavanagh, Andrea D. Smith, Lucilla Poston, Sara L. White

**Affiliations:** ^1^ Department of Women and Children's Health, School of Life Course Sciences, King's College London, London, United Kingdom.; ^2^ Department of Mathematics and Statistics, University of Strathclyde, Glasgow, United Kingdom.; ^3^ Department of Behavioural Science and Health, University College London, London, United Kingdom.

**Keywords:** coronavirus, COVID-19, preconception, pregnancy planning, survey, women's health care

## Abstract

***Background:*** Our understanding of how the coronavirus disease 2019 (COVID-19) pandemic has impacted decision-making for women planning to conceive is unclear. We aimed to investigate how the COVID-19 pandemic has influenced pregnancy planning behaviors.

***Methods:*** An online questionnaire of closed- and open-ended questions was utilized to capture pregnancy planning behaviors and reported behavioral changes during the COVID-19 pandemic in women planning pregnancy between January and July 2020. Closed-ended questions were analyzed quantitatively, and thematic framework analysis was utilized for open-ended responses.

***Results:*** A total of 504 questionnaires were included for analysis. The majority of respondents lived in the United Kingdom. Ninety-two percent of the women were still planning a pregnancy but over half (*n* = 267) reported that COVID-19 had affected their plans, with 72% of these (*n* = 189) deliberately postponing pregnancy. Concerns were predominantly over changes in antenatal care, but also fear of adverse effects of the virus on mother and baby. From the thematic analysis (*n* = 37), lack of services to remove contraceptive devices and provide fertility treatment were also cited. In contrast, 27% (*n* = 71) reported bringing their pregnancy plans forward; common themes included recalibration of priorities and cancelled or changed plans.

***Conclusions:*** The COVID-19 pandemic influenced pregnancy-planning behaviors with many women reporting postponement of pregnancy. These alterations in behavior could impact the health and wellbeing of women planning pregnancy while having important implications for health care services worldwide. Continued provision of family planning and fertility services should be ensured to mitigate the effect of future outbreaks or pandemics.

## Introduction

The severe acute respiratory syndrome coronavirus (SARS-CoV-2) emerged in December 2019 and rapidly spread across the globe.^1^ Although the effects of the coronavirus disease 2019 (COVID-19) pandemic on pregnancy outcomes have been the focus of attention and are increasingly reported,^2–4^ our understanding of how COVID-19 has impacted, and will impact on decision-making for women planning to conceive is limited. To address this, quantitative and qualitative data are required to devise evidence-based and context-appropriate mitigation strategies.

Achieving readiness for pregnancy through appropriate preconception planning is key to optimizing pregnancy outcomes, with important consequences for maternal and child health.^5^ Surveys undertaken using the London Measure of Unplanned Pregnancy questionnaire^6^ suggest that pregnancy planning is universally common,^7–9^ and may be influenced by a range of social, medical, and environmental factors.^5^ In the wake of the COVID-19 pandemic, and the associated lockdown, these factors likely substantially changed for the majority of women planning to conceive. Moreover, many aspects of antenatal and birth care moved away from hospital face-to-face visits toward virtual/community-based care.^10^

The aim of this study was to examine how the COVID-19 pandemic influenced pregnancy planning in women who used a digital online tool between January and July 2020 to access information on preconception health. Our data indicate that COVID-19 influenced pregnancy planning, causing women to both postpone pregnancy and, interestingly, bring plans forward. Postponement was driven not only by concerns regarding the health effects of COVID-19, but also by anxiety regarding the impact of the pandemic on antenatal care. Together, these data suggest a need for public health messaging directed at women planning pregnancy during the unprecedented social upheaval driven by COVID-19.

## Methods

### Study design and population

We conducted a questionnaire-based online survey by contacting women who had recently filled out a digital preconception tool (Tommy's “Planning for Pregnancy” tool. Facebook social media adverts were used to target women planning pregnancy. This included mainstream targeting (very wide targeting of women, age 16–45), women from minority ethnic groups, low-income women, and those with a high body mass index (BMI). For the purposes of this study, women who had agreed to such contact were approached by email if they had utilised the preconception tool between January and July 2020 and had provided their email address. The email contained a link inviting the woman to complete an online questionnaire that assessed the impact of the pandemic on her pregnancy plans.

### Data collection

The online questionnaire (Google forms) included a combination of closed- and open-ended questions to capture pregnancy planning and subsequent changes as a result of the COVID-19 pandemic **(**Supplementary Data S1**)**. The questionnaire was developed in consultation with multidisciplinary academics and health professionals in the field of women's health. Women who were planning a pregnancy were asked how the COVID-19 pandemic had affected their plans; if they had postponed their pregnancy, brought their plans forward, or plans were unchanged. The respondents could select a listed response option or enter a free text comment. For women who were pregnant, they were asked through an open-ended question how the COVID-19 pandemic had affected their pregnancy. Participants were prompted to elaborate on both positive and more difficult aspects in relation to the open-ended questions.

Demographic information requested included age (18–24, 25–34, 35–40, 41+ years), ethnicity (white, black, Asian, mixed, and other), highest educational achievement (GCSE or equivalent, A level or equivalent, degree or equivalent, none), country of residence, and self-reported weight and height to calculate BMI (weight/height; kg/m^2^) according to the World Health Organization classification: underweight (≤18.5 kg/m^2^); normal weight (18.5–24.9 kg/m^2^); overweight (25–29.9 kg/m^2^); and obese (≥30 kg/m^2^).^11^

### Data and statistical analysis

Data were quality checked for entry errors. Duplicate entries were removed if there was clear evidence of repeated submissions (nonblank identical free text responses). Proportions and numbers responding to each were reported. Respondents were dichotomized according to country of residence; into participants residing in the United Kingdom (U.K. respondents), or all other countries (non-U.K. respondents). Those preferring not to report their country were included in the non-U.K. group. Statistical analysis was conducted in R version 4.0.2 (R Core team, 2016).

Open responses were analyzed using framework analysis.^12^ The responses were reviewed, and a list of themes developed. Next, the responses were coded and combined into the final framework. We narratively report on prominent themes arising from the framework analysis.

## Results

### Characteristics of women who responded to the survey

Between January and July 2020, 5,157 women completed the Tommy's online tool and left their email address. Between July 3 and 23, 2020, the present questionnaire was completed by 520 respondents giving a response rate of 10%. Following removal of 16 duplicates, 504 questionnaires were available for analysis. The majority of respondents resided in the United Kingdom (*n* = 335; 65.5%). Eighty-five percent of respondents were between 25 and 40 years of age. The respondents were predominantly of white (64.7%) and black (21.2%) ethnic groups. Sixty-nine percent were educated to degree level while over a quarter were overweight (26%) and 18.3% obese **(**Table 1**)**.

**Table 1.** Descriptive Characteristics of the Respondents

	No. of respondents^a^ (*N* = 504)	
	*N*	%
Age group, years		
18–24	50	9.9
25–34	324	64.3
35–40	106	21
41+	20	4
Prefer not to say	4	0.8
Ethnicity		
Asian	34	6.7
Black	107	21.2
Mixed	20	4
Other	9	1.8
Prefer not to say	8	1.6
White	326	64.7
Level of education		
A level or equivalent	76	15.1
Degree or equivalent	348	69
GCSE or equivalent	40	7.9
None	5	1
Prefer not to say	35	6.9
BMI^a^		
Underweight	12	3.1
Normal	205	52.7
Overweight	101	26
Obese	71	18.3
Country		
England	294	58.3
Scotland	22	4.4
Wales	12	2.4
Northern Ireland	7	1.4
Non-United Kingdom^b^	168	33.5

^a^ For BMI the total number of respondents who returned values to calculable BMI was 389 in total, 286 in the United Kingdom and 103 in the non-United Kingdom.

^b^ Respondents (including 1.6% who did not disclose their country) from other 34 countries—South Africa, Nigeria, United States, Republic of Ireland, Kenya, Australia, India, Ghana, Canada, Pakistan, Uganda, United Arab Emirates, Zambia, Honduras, Indonesia, Jamaica, Kazakhstan, Russian Federation, South Sudan, Switzerland, Trinidad and Tobago, Azerbaijan, Barbados, Germany, Greece, Guyana, Italy, Malawi, Namibia, the Netherlands, New Zealand, Saint Vincent and the Grenadines, Thailand, and Zimbabwe.

BMI, body mass index.

### Impact of the COVID-19 pandemic on women who were planning a pregnancy

Ninety-two percent of respondents reported they were still planning a pregnancy since submission of the original preconception questionnaire. Over half (*n* = 267, 53%) reported that COVID-19 had affected their pregnancy plans, with 71.9% of these reporting deliberate postponement of pregnancy. Conversely, 27% had brought their plans for pregnancy forward and 1.1% reported that they were no longer planning a pregnancy as a result of the pandemic **(**Table 2**)**.

**Table 2.** Impact of the COVID-19 Pandemic on Women Planning Pregnancy

Question	Total responders, *N*^a^	Response	No. of responding	%	95% CI
Since filling out the Tommy's online tool are you still planning a pregnancy?	504	No	39	7.7	5.7–10.4
		Yes	465	92.3	89.6–94.3
Has COVID-19 affected your plans?	504	No	237	47	42.7–51.4
		Yes	267	53	48.6–57.3
How has COVID-19 affected your plans?	263	I am no longer planning a pregnancy at all	3	1.1	0.4–3.3
		I have brought forward my plans for pregnancy	71	27	22–32.7
		I have postponed pregnancy	189	71.9	66.1–77.0
When do you plan to start trying again?	191^b^				
		2–6 months	51	26.7	20.2–34.6
		6+ months	42	22	14.6–27.8
		Not until a vaccine	8	4.2	0–3.9
		Within 2 months	90	47.1	43.9–60.2

^a^ Number of individuals who completed the question. Not all individuals completed all the questions, which they were eligible to, based on their previous responses.

^b^ All the 189 individuals who responded to the question “How has COVID 19 affected your plans?” answered the question “When do you plan to start trying again?.” In addition, two individuals indicated that COVID-19 had affected their plans but did not respond as to why in the previous question. Their results are included in the responses to this question, as their free text responses indicate that COVID-19 had impacted on fertility/medical investigations and consequently they had postponed pregnancy.

CI, confidence interval; COVID-19, coronavirus disease 2019.

For those reporting postponed pregnancy (*n* = 189), selection or one or more from a list of reasons was available or free text could be entered. From the list of reasons, the most commonly cited were “concern about change in pregnancy care” and “concern about the effects of the virus on mum or baby” **(**Table 3**)**. Prominent themes from the open comments (*n* = 37) included lack of services to remove contraceptive devices or provide fertility treatment services. Reduced support from health care professionals, access to services, and concerns over future employment security were additional reasons for postponing pregnancy. Women commonly reported that they were unable to get a coil or implant removed, they were unable to get referrals to fertility clinics, fertility appointments were cancelled, while postponement of treatment for conditions such as endometriosis and polycystic ovaries were also reported. Miscarriages were stated by some women as a reason to postpone pregnancy with negative experiences of attending hospital services alone reported.

**Table 3.** Survey Responses of Women Who Deliberately Postponed Pregnancy as a Result of the COVID-19 Pandemic

Response	*n*	%	95% CI
Question: Why have you chosen to postpone pregnancy? (select as many as you wish)			
Concern about change in pregnancy care during pandemic	106	55.2	48.1–62.1
Concern about the effects of the virus on yourself or your baby	101	52.6	45.6–59.5
You consider yourself to be of high-risk ethnicity for the virus	9	4.7	2.5–8.7
You have an underlying health condition	16	8.3	5.2–13.1
You are living with vulnerable family members	11	5.7	3.2–10
Other (free text reasons)	53	27.6	21.8–34.3

For those who had postponed pregnancy, almost half (47.1%) reported that they planned to start trying again within 2 months, 26.7% reported between 2 and 6 months, 22% reported 6 months or over, and 4.2% reported that they did not plan to start trying again before receiving a vaccine.

For women who had brought their pregnancy plans forward (*n* = 71), common themes from the open comments included cancelled and change of plans such as wedding, holiday, and work plans allowing for an earlier pregnancy. Some women reported recalibration of priorities, including time to evaluate what is important in life and deciding not to wait any longer. Some women reported age as a reason to “get on” with trying if plans were cancelled or priorities changed. Working from home and more flexibility were also reported as reasons to bring pregnancy plans forward with women reporting a better work/life balance. Current disruption and future uncertainties about the state of the world also appeared to increase perceived urgency to conceive.

Of respondents who reported that COVID-19 had not affected their pregnancy plans (*n* = 237, 47%), 84.8% reported that they were still trying to conceive and 13.5% reported that they were pregnant (data not shown).

### Impact of the COVID-19 pandemic on women who were pregnant

For respondents who were pregnant (*n* = 30), themes included the negative impact of the pandemic owing to changed antenatal care/lack of support, partners unable to attend antenatal appointments, and increased anxiety and stress about changed birthing plans. Positive experiences were also reported with themes including being able to work from home, and less travelling and commuting that had freed up time for self-care.

## Discussion

### Main findings

Maternal health behaviors and environmental influences in the weeks and months before conception can influence pregnancy outcomes, and the longer term health of the mother and child. The benefits of preconception and interconception care for both women and men are increasingly appreciated as a means to improve long-term health.^5^ Conversely, negative influences on pregnancy planning could be detrimental to population health. Here, we report that the COVID-19 pandemic has led to changes in pregnancy planning with a large proportion of women reporting deliberate postponement of their pregnancy plans.

Our findings are consistent with U.K. data suggesting a decrease in antenatal booking appointments between April and September 2020 compared with the same period in 2019.^13^ This study provides a novel insight into pregnancy planning of women during the COVID-19 pandemic and may underpin the trend toward a decrease in pregnancy rates. Women highlighted anxiety around the effects of the virus, the impact on antenatal services, and lack of access for removal of contraceptive devices and fertility services as the main reasons for pregnancy postponement, all of which may have contributed to the decline in pregnancy rate in 2020. Should further pandemics and associated social distancing occur in the future, a pragmatic approach is warranted to ensure women can access health services in a safe environment while being provided with appropriate support and information from health care professionals. Services to remove and insert contraceptive devices and fertility treatment continuation should be considered. Furthermore, appropriate and safe measures should be taken to enable women to have in-person support while attending antenatal or other health services.

Although our results would also seem to contrast with mainstream media coverage predicting a “COVID-19 baby boom,” our findings are in line with reports that suggest that in times of uncertainty, longer term reproductive commitments are postponed.^14^ Moreover, global fertility trajectories owing to the COVID-19 pandemic and associated social distancing measures may differ depending on economic status. Factors such as a society's development, demographic transition, access to contraception, and availability of assisted reproductive technology through fertility services may have a differential effect on conception rates. In high-income countries, conception rates may decrease as a result of a negative change in work/life balance and reduced access to fertility services, and an increase in economic loss and uncertainty.^15^ This is supported by the responses of women delaying pregnancy in the present study who highlighted a reduction in fertility service access and concerns over current and future employment status.

Optimal prepregnancy care includes folic acid supplementation, smoking cessation, stopping alcohol consumption, and achievement/maintenance of a healthy weight.^5^ Previous reports have indicated that adequate pregnancy planning improves some of these behaviors. Data from the U.K. Millennium Cohort Study showed that women (*n* = 18,178) who planned their pregnancy were less likely to smoke, and more likely to have babies with a healthy birth weight and less prematurity compared with unplanned pregnancies.^16^ Our data indicate that the COVID-19 pandemic may lead to pregnancy postponement, increased pregnancy planning, and paradoxically, may have the unexpected benefit of driving positive health behaviors during the preconception period. Conversely, the pandemic might potentially lead to sedentary or suboptimal health behaviors that detrimentally impact pregnancy outcomes.^17^

Although there was an increase in mortality and morbidity documented in pregnant women infected with SARS-CoV-1,^18^ as evidence emerges, severe maternal illness seems relatively uncommon in relation to SARS-CoV-2.^19^ However, in the period of data collection, the effects of SARS-CoV-2 on pregnancy outcome was unclear and this may have impacted pregnancy planning. Many women reported that they postponed pregnancy because of concerns regarding the health effects of the virus on mother and child, which is similar to reports of Brazilian women during the Zika virus outbreak of 2015–2016,^20^ and possibly because of the wide publicity over Zika and pregnancy. Conversely, in this study, a significant proportion of women contacted brought pregnancy plans forward owing to recalibration of priorities for themselves and their family. Together these data indicate that the pandemic has impacted pregnancy timing; however, the directionality of these effects are dictated largely by personal circumstances.

Because hospital attendance may place obstetric patients at additional risk of contracting SARS-CoV-2, there has been an increased emphasis on virtual consultation; which may give the perception that care will be suboptimal. For pregnant women with suspected/confirmed infection, further disruption includes delay of nonurgent routine appointments (such as growth scans, antenatal community, or secondary care appointments), because of the need for a period of self-isolation.^10^ These types of considerations also influenced women's decision-making with regard to pregnancy planning owing to concern over pregnancy care. This highlights the value of developing accessible and reliable virtual information platforms to ensure that up-to-date information on pregnancy planning and health is made readily available, even in emergencies like a pandemic.

Several women reported disruption in routine clinical activity to remove contraceptive devices and cessation of fertility treatments. In line with our findings, a recent U.S. study estimated the impact of the postponement of fertility treatment on birth rates, particularly affecting older women.^21^ In normal circumstances, treatment for infertility is associated with emotional distress^22^ and should similar quarantines occur again for any reason in the future, a balanced approach to maintain services and support in a safe environment should be a priority. This same strategy should be used for maintaining contraception and sexual health services for promotion of safe family planning.

### Strengths and limitations

As data indicating the impact of the COVID-19 pandemic on pregnancy emerges, to our knowledge this is the first report on the effect of COVID-19 on women actively planning pregnancy. Using both a quantitative and qualitative approach, this study comprehensively evaluated the impact of the pandemic on pregnancy planning behaviors. The inclusion of open-ended questions in the questionnaire provides unprecedented and nuanced insights on the diverse ways the COVID-19 pandemic influenced pregnancy-planning behaviors.

The presented findings provide a snapshot of women's pregnancy planning behaviors during a specific phase of the pandemic only. Social, environmental, and psychological states will have changed over the course of the pandemic and these temporal changes have not been tracked by this study. A further limitation of this study is the self-report nature of the data included and representativeness of the wider population of women. The women reported a high level of education, which may be owing to social desirability bias or biases in the recruitment method. In Stephenson et al., which aimed to determine the extent to which women plan and prepare for pregnancy in the United Kingdom, 66% of women in early pregnancy reported being educated to degree level, which is consistent with the women represented in our findings.^23^ In addition, in a recent Australian study describing lifestyle behaviors of women planning pregnancy, 60% of women were educated to university level.^24^ In contrast, Inskip et al. examined the adherence of U.K. women planning pregnancy to lifestyle recommendations and found that 21% of women were educated to degree level.^25^ The majority of women in this study were white, which is also consistent with Stephenson et al.^23^ The presented findings, therefore, need to be interpreted with this potential limitation of generalizability in mind.

### Interpretation and implications for practice

Our findings suggest that the COVID-19 pandemic has influenced women's pregnancy planning behaviors. Women reported delaying pregnancy because of concerns about pregnancy care and the effects of the virus. Additional themes emerged including suspension of routine clinical and fertility services, which led to postponement. Until a vaccine is accessible for all, continued access to women's health care services and clear public health messages surrounding the virus must be in place to support those planning a pregnancy during this crisis.

## Conclusion

The COVID-19 pandemic has significantly influenced pregnancy-planning behaviors that may have lasting impact, positive or negative, on the health and well-being of women and impact health care services worldwide. Understanding short-term impacts of COVID-19 on pregnancy planning behaviors provides immediate insights to inform evidence-based guidance for responding to the current pandemic to mitigate detrimental mental and physical health impacts on women planning for an imminent pregnancy. Simultaneously, these findings add to the emerging evidence that health care systems maintain continued provision of family planning and fertility services should future outbreaks or pandemics occur.

## Supplementary Material

Supplemental data

## Abbreviations Used

BMI: body mass index
CI: confidence interval
COVID-19: coronavirus disease 2019
SARS-CoV-2: severe acute respiratory syndrome coronavirus
