# Supplementary material for: The Impact of the COVID-19 Pandemic on Pregnancy Planning Behaviors
Source: Womens Health Rep (New Rochelle). 2021 Mar 23;2(1):71–7. doi: 10.1089/whr.2021.0005 (PMC8006747; doi:10.1089/whr.2021.0005)
Supplement: Supplemental data [file Supp_DataS1.pdf]

Planning for pregnancy and COVID-19

* Required

1. 1. Since filling out the Tommy's online tool are you still planning a pregnancy? *

Mark only one oval.

- Yes
- No

2. 2. Has COVID-19 affected your plans? *

Mark only one oval.

- Yes
- No *Skip to question 8*

Plans affected by COVID-19

3. 3. How has COVID-19 affected your plans?

Mark only one oval.

- I have postponed pregnancy
- I have brought forward my plans for pregnancy *Skip to question 12*
- I am no longer planning a pregnancy at all *Skip to question 7*

Pregnancy postponed

4. 4. Why have you chosen to postpone pregnancy?

Check all that apply.

- [ ] Concern about change in pregnancy care during pandemic
- [ ] Concern about the effects of the virus on yourself or your baby
- [ ] You consider yourself to be of high risk ethnicity for the virus
- [ ] You have an underlying health condition
- [ ] You are living with vulnerable family members
- [ ] Your financial situation (money) has changed
- [ ] Other - please give additional reasons below.

5. What are your other reasons for postponing pregnancy?

____

____

____

____

____

6. When do you plan to start trying again

Mark only one oval.

- ( ) Within 2 months *Skip to question 13*
- ( ) 2 - 6 months *Skip to question 13*
- ( ) 6+ months *Skip to question 13*
- ( ) Not until a vaccine *Skip to question 13*

No longer wish to conceive

7. How has COVID-19 caused you to decide not to try to become pregnant at all?

Skip to question 13

Plans unaffected by COVID-19

8. COVID-19 has not affected your plans because:

Mark only one oval.

- You are still trying to get pregnant *Skip to question 13*
- You are currently pregnant, have recently been pregnant or recently given birth *Skip to question 9*
- You have had a baby (prior to COVID-19) *Skip to question 13*
- For other reasons a baby is not on your radar *Skip to question 13*

Effect of COVID-19 on pregnancy

9. How has COVID-19 affected your pregnancy? (please note both good and more difficult things)

10. How did COVID-19 affect the birth of your baby if applicable? (please note both good and more difficult things)

11. How did COVID-19 affect you after your baby was born if applicable? (please note both good and more difficult things)

Skip to question 13

Bringing plans forward

12. Why have you brought plans for pregnancy forward?

Demographics

13. What was your age at your last birthday? *

Mark only one oval.

- 18 - 24
- 25 - 34
- 35 - 40
- 41+
- Prefer not to say

14. We wish to record your weight - please state the unit you wish to record this in *

Mark only one oval.

- Kilograms (Kg) *Skip to question 15*
- Pounds *Skip to question 16*
- Stones *Skip to question 17*
- I prefer not to record my weight *Skip to question 22*

Weight (Kg)

15. What is your weight now (or pre pregnancy if applicable) in Kg? *

____

Skip to question 18

Weight (Lbs)

16. What is your weight now (or pre pregnancy if applicable) in Pounds

____

Skip to question 18

Weight (Stone)

17. What is your weight now (or pre pregnancy if applicable) in Stones

____

Height

18. We wish to record your height - which unit to you wish to record this in? *

Mark only one oval.

- ( ) Centimeters *Skip to question 19*
- ( ) Feet and inches *Skip to question 20*
- ( ) I prefer not to record my height *Skip to question 22*

Height (Cm)

19. What is your height in Centimetres (cm)? *

____

Skip to question 22

Height (Ft, Inch)

20. What is your height in Feet

____

21. and inches

____

Demographics cont.

22. What ethnic group do you belong to? *

Mark only one oval.

- Black
- Asian
- White
- Mixed
- Other
- prefer not to say

23. What is your highest educational achievement? *

Mark only one oval.

- None
- GCSE or equivalent
- A level or equivalent
- Degree or equivalent
- prefer not to say

24. In which Country do you live *

Mark only one oval.

- Prefer not to say
- England
- Scotland
- Ireland (Republic)
- Northern Ireland
- Wales
- Afghanistan
- Albania
- Algeria
- Andorra
- Angola
- Antigua & Deps
- Argentina
- Armenia
- Australia
- Austria
- Azerbaijan
- Bahamas
- Bahrain
- Bangladesh
- Barbados
- Belarus
- Belgium
- Belize
- Benin
- Bhutan
- Bolivia
- Bosnia Herzegovina
- Botswana
- Brazil
- Brunei
- Bulgaria

- Burkina
- Burundi
- Cambodia
- Cameroon
- Canada
- Cape Verde
- Central African Rep
- Chad
- Chile
- China
- Colombia
- Congo
- Congo (Democratic Rep)
- Costa Rica
- Croatia
- Cuba
- Cyprus
- Czech Republic
- Denmark
- Djibouti
- Dominica
- Dominican Republic
- East Timor
- Ecuador
- Egypt
- El Salvador
- Equatorial Guinea
- Eritrea
- Estonia
- Ethiopia
- Fiji
- Finland
- France
- Gabon
- Gambia

- Georgia
- Germany
- Ghana
- Greece
- Grenada
- Guatemala
- Guinea
- Guinea-Bissau
- Guyana
- Haiti
- Honduras
- Hungary
- Iceland
- India
- Indonesia
- Iran
- Iraq
- Israel
- Italy
- Ivory Coast
- Jamaica
- Japan
- Jordan
- Kazakhstan
- Kenya
- Kiribati
- Korea North
- Korea South
- Kosovo
- Kuwait
- Kyrgyzstan
- Laos
- Latvia
- Lebanon
- Lesotho

- Liberia
- Libya
- Liechtenstein
- Lithuania
- Luxembourg
- Macedonia
- Madagascar
- Malawi
- Malaysia
- Maldives
- Mali
- Malta
- Marshall Islands
- Mauritania
- Mauritius
- Mexico
- Micronesia
- Moldova
- Monaco
- Mongolia
- Montenegro
- Morocco
- Mozambique
- Myanmar (Burma)
- Namibia
- Nauru
- Nepal
- Netherlands
- New Zealand
- Nicaragua
- Niger
- Nigeria
- Norway
- Oman
- Pakistan

- Palau
- Panama
- Papua New Guinea
- Paraguay
- Peru
- Philippines
- Poland
- Portugal
- Qatar
- Romania
- Russian Federation
- Rwanda
- St Kitts & Nevis
- St Lucia
- Saint Vincent & the Grenadines
- Samoa
- San Marino
- Sao Tome & Principe
- Saudi Arabia
- Senegal
- Serbia
- Seychelles
- Sierra Leone
- Singapore
- Slovakia
- Slovenia
- Solomon Islands
- Somalia
- South Africa
- South Sudan
- Spain
- Sri Lanka
- Sudan
- Suriname
- Swaziland

- Sweden
- Switzerland
- Syria
- Taiwan
- Tajikistan
- Tanzania
- Thailand
- Togo
- Tonga
- Trinidad & Tobago
- Tunisia
- Turkey
- Turkmenistan
- Tuvalu
- Uganda
- Ukraine
- United Arab Emirates
- United States
- Uruguay
- Uzbekistan
- Vanuatu
- Vatican City
- Venezuela
- Vietnam
- Yemen
- Zambia
- Zimbabwe

This content is neither created nor endorsed by Google.

Google Forms
